# Thermoplastic Micromodel Investigation of Two-Phase Flows in a Fractured Porous Medium

**DOI:** 10.3390/mi8020038

**Published:** 2017-01-26

**Authors:** Shao-Yiu Hsu, Zhong-Yao Zhang, Chia-Wen Tsao

**Affiliations:** 1 Department of Bioenvironmental System Engineering, National Taiwan University, Taipei 10617, Taiwan; syhsu@ntu.edu.tw; 2 Department of Mechanical Engineering, National Central University, Taoyuan 32001, Taiwan; stargold1224@gmail.com

**Keywords:** thermoplastic, micromodel, cyclic olefin copolymer, UV/Ozone bonding, residual trapping, flow visualization

## Abstract

In the past few years, micromodels have become a useful tool for visualizing flow phenomena in porous media with pore structures, e.g., the multifluid dynamics in soils or rocks with fractures in natural geomaterials. Micromodels fabricated using glass or silicon substrates incur high material cost; in particular, the microfabrication-facility cost for making a glass or silicon-based micromold is usually high. This may be an obstacle for researchers investigating the two-phase-flow behavior of porous media. A rigid thermoplastic material is a preferable polymer material for microfluidic models because of its high resistance to infiltration and deformation. In this study, cyclic olefin copolymer (COC) was selected as the substrate for the micromodel because of its excellent chemical, optical, and mechanical properties. A delicate micromodel with a complex pore geometry that represents a two-dimensional (2D) cross-section profile of a fractured rock in a natural oil or groundwater reservoir was developed for two-phase-flow experiments. Using an optical visualization system, we visualized the flow behavior in the micromodel during the processes of imbibition and drainage. The results show that the flow resistance in the main channel (fracture) with a large radius was higher than that in the surrounding area with small pore channels when the injection or extraction rates were low. When we increased the flow rates, the extraction efficiency of the water and oil in the mainstream channel (fracture) did not increase monotonically because of the complex two-phase-flow dynamics. These findings provide a new mechanism of residual trapping in porous media.

## 1. Introduction

When applying engineering processes to porous media involving two-phase flows, e.g., enhanced oil recovery, geological carbon sequestration, water infiltration, and groundwater remediation, a fractured pore structure is commonly observed. To understand the fluid interactions in a fractured porous material, a uniform pore-pattern embedded with a single macropore channel is commonly used to represent the pore geometry of the fractured porous medium [1,2,3,4]. A thorough conceptual and quantitative understanding of the flow behavior of fluids in pore spaces forms the basis of multiphase flow in porous materials. These subsurface multiphase flows are of interest to hydrologists, agricultural/environmental engineers, and petroleum engineers. Nevertheless, since most soils and geomaterials are not transparent, visualizing their flow behavior in porous materials is a long-term persistent challenge.

Micromodels are one of the commonly used tools for investigating and visualizing physical, chemical, and biological processes at a small scale in two or three dimensions [5]. Karadimitriou and Hassanizadeh [5] defined a micromodel as “an artificial representation of a porous medium made of a transparent material.” A micromodel usually comprises an artificial pore structure of connected pores, whose shapes are designed to represent the simplified geometry of a geomaterial. The geometries of the early micromodels were mostly simple and regular [6,7]. Since the 1980s, micromodel geometry has become more complicated, and various sizes of pores and channels have been computer-generated based on statistical distributions and fractal patterns [8,9]. Some irregular patterns that have properties similar to real porous media have been generated using the Delaunay triangulation [10]. Such patterns can also be designed for studying flow behaviors in pore geometries that are present either in natural porous materials or in theoretical models [1,3,11].

Micromodels for porous media are usually fabricated using glass plates packed with glass beads [12,13]. In addition, glass [14], silicon wafer [15], and photoresist [16] surfaces have been used to construct micromodels. The material cost and, in particular, the microfabrication-facility cost for making a glass or silicon-based micromodel are high; this creates obstacles for researchers investigating two-phase-flow behavior in porous media. Polymers are low-cost materials and are therefore an attractive alternative materials for microscale geomaterial models. Various polymer materials with different mechanical, chemical, and optical properties are available in the market; more importantly, the facility costs for making polymer micromodels are much lower than those for glass or silicon micromodels. Polymer materials have been proposed in the microfluidics community since the late 1990s [17] and are being increasingly used since then owing to their low cost and disposability. Polymer materials such as thermoplastics, polycarbonate (PC), polystyrene (PS), polymethylmethacrylate (PMMA), and cyclic olefin copolymer (COC) or polydimethylsiloxane (PDMS) elastomers are commonly used in microfluidics. Rigid thermoplastic materials are preferable for porous micromodels because they have better resistance to surface infiltration and deformation than PDMS [18]. An artificial pore structure can be fabricated via replication using a thermoplastic material. Polymer-replication processes such as injection molding [19,20], hot embossing [21,22,23], and roller printing [24,25] require a master micromold to produce the inverted polymer replicas for mass production. After replication, bonding is necessary with another cover plate to create an enclosed microchannel. Various thermoplastic bonding methods have been reported for sealing the microchannel [26]. Thermoplastic bonding is one of the most simple and straightforward thermoplastic processes [23]; however, it imparts a low bonding strength. Therefore, high-strength bonding techniques using materials such as solvents [27], adhesives [28], UV/Ozone [29], O_2_ plasma [30], as well as welding [31] have been reported. Each polymer-replication or bonding technique presents specific advantages as well as process limitations for different applications. Therefore, appropriate selection of the polymer microfabrication process is important while developing micromodels for geo-fluid experimental usage. 

Two-phase-flow experiments in a well-manufactured micromodel with a fractured pattern are still limited. A challenge in using polymer materials for representing natural porous materials is the polymers’ surface wettability. The water contact angle of soil cracks is approximately 20°–30°; however, it varies under different conditions [32,33,34]. Generally, natural porous materials are hydrophilic. Unlike glass or silicon substrates, polymer substrates such as PC, PMMA, and COC usually exhibit weak hydrophobicity, which may not be suitable for representing hydrophilic geomaterials. A hydrophilic surface coating may be used in a porous polymer micromodel. Normally, this coating is temporary, and the aqueous-phase coating reagent may not be uniformly filled and removed from the microchannel for homogeneous coating, especially in our high-density porous micromodel design. In this study, we demonstrate the use of COC, a thermoplastic material, in geo-fluid experimental micromodel investigations. In addition, we fabricate an artificial pore structure via hot embossing and use UV/Ozone for bonding as well as for controlling surface wettability. Furthermore, we perform two-phase-flow micromodel experiments in a fractured pattern and investigate the spatial distribution of oil and groundwater subjected to injection and extraction rates.

## 2. Experimental Section 

### 2.1. Materials and Reagent

The materials used for fabricating the micromodels include COC, silicon wafer, SU-8 resist, surgical needle, acetone (ACE), and isopropyl alcohol (IPA). COC 8007 granules were purchased from TOPAS Advanced Polymers Inc. (Florence, KY, USA). The COC granules were injected into custom-made stainless-steel molds to fabricate COC substrates with a diameter of 7 cm and a thickness of 2 mm. A P-type (100) silicon wafer with a diameter of 10 cm was purchased from Summit-Tech Resource Corp. (Hsinchu, Taiwan). SU-8 3050 and SU-8 developer were purchased from MicroChem Corp. (Westborough, MA, USA). Surgical needles (SC20/15, LS20) were purchased from Instech Laboratories Inc. (Plymouth Meeting, PA, USA). Medical-grade tubing (Tygon S-50-HL) was purchased from Saint-Gobain Performance Plastics Corp. (Akron, OH, USA). ACE and IPA were purchased from Sigma-Aldrich (St. Louis, MO, USA).

### 2.2. Contact Angle Measurement

The contact angle of the COC surface was measured using a custom-made optical goniometer comprising a high-resolution digital camera (Canon EOS 450D/TAMRON Macro 90 mm F2.8 lens), a precision stage, and an optical light source. To measure the contact angle, a 5-μL water droplet was placed on the COC surface using a pipette; then, a side-view image of the microdroplet was taken using a digital camera. The contact angle was determined by measuring the angle formed between the microdroplet tangent line and the COC surface on the basis of microdroplet images obtained using AutoCAD 2013 (Autodesk, Inc., Mill Valley, CA, USA).

### 2.3. Micromold Fabrication

Fabrication of the SU-8 micromold started with cleaning silicon water with ACE, IPA, deionized (DI) water, and N_2_ blow-drying followed by hot baking at 130 °C for 15 min (Super-Nuova, Thermo Scientific Inc., Ocala, FL, USA). Spin-coating with a thin hexamethyldisilazane (HMDS) was applied to remove the moisture from the silicon surface; subsequently, spin-coating was performed with SU-8 3050 at 700 rpm for 30 s and at 1200 rpm for 40 s (SPC-703, Yi Yang Co., Taoyuan, Taiwan) to apply a 100-μm SU-8 layer on the silicon substrate. Then, soft baking was performed at 110 °C for 15 min. The mask was aligned and then exposed to UV (AGL100 UV Light source, M&R Nano Technology Co., Taoyuan, Taiwan) for 90 s. Then, the UV-exposed SU-8 substrate was hard-baked at 95 °C for 5 min, and the SU-8 micromold was developed in the SU-8 developer within 2–3 min. The SU-8 micromold was rinsed clean with IPA, DI water, and N_2_ blow drying. Finally, a hard-baking cure at 130 °C was applied for 8 h to enhance the SU-8 micromold’s mechanical strength for the hot-embossing process.

### 2.4. Micromodel Design and Experimental Setup 

**Figure 1:**
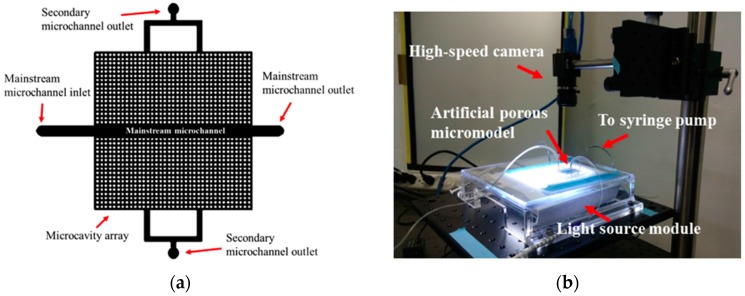
(**a**) Artificial porous micromodel design to mimic a natural fractured porous material. (**b**) Photograph of the porous micromodel experimental setup.

The artificial porous micromodel design is shown in Figure 1. To mimic a natural fractured porous material, the micromodel device comprises a 2.6-mm-wide and 50-mm-long microchannel (mainstream microchannel) in the middle of a uniform 30 mm × 30 mm pore pattern containing 1332 pieces of microcavities. Each microcavity was 500 μm in diameter and 100 μm in depth. The microcavities were separated by a 200-μm distance (see Figure 1a). 

The artificial pattern represents a two-dimensional cross-section of a fractured pore material. The inlet and outlet of the mainstream microchannel represent the injection and extraction points of water and oil during groundwater remediation or enhanced oil recovery, respectively. The secondary outlets represent another parallel fracture connected to air or another extraction well away from the main fracture. They allow the displaced fluid to flow in the direction perpendicular to the main flow direction.

The experimental setup of the micromodel experiment comprised a COC micromodel, a syringe pump (KD Scientific Legato 100), a high-speed camera (Point Grey Flea3, CMOS sensor, maximum resolution: 1280 × 1024 with maximum frame rate of 150 fps), and a background light source (Figure 1b). Images of the entire micromodel were taken with the high-speed camera to record the changes in the two fluid distributions during both the imbibition and drainage processes. The left inlet of the mainstream of the micromodel was connected to the syringe pump, which provided a constant-flow-rate boundary condition. The secondary microchannel outlets connected to atmospheric air provided a constant-pressure boundary condition. The secondary outlets represented another parallel fracture connected to air or another extraction well away from the main fracture.

## 3. Results and Discussion

### 3.1. Polymer Micromodel Fabrication

**Figure 2:**
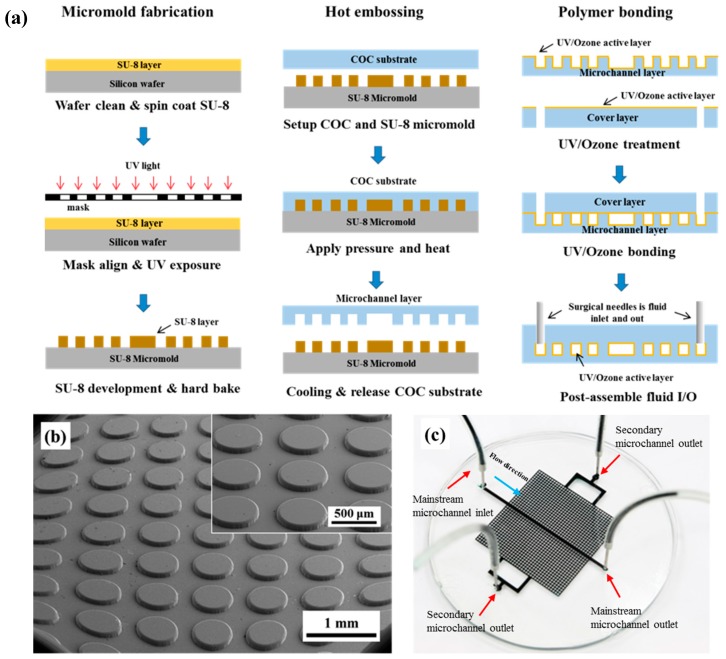
(**a**) Schematic of the micromodel fabrication flow chart. (**b**) 40× fisheye SEM image of the SU-8 micromold showing microcavity arrays. The top-right corner shows an enlarged 100× SEM image. (**c**) Micromodel with black-food-dye injection.

Polymer selection is the first important step in determining the success of microfluidic-chip fabrication for an artificial porous micromodel. In our design, because the micromodel comprises 1332 pieces and 500-μm microcavities, the hydraulic resistance of the microchannel is high. Thus, a high pumping pressure is required to drive the fluid flow. Moreover, a good mechanical strength is required; therefore, an elastomer material such as PDMS is not preferred in this study. A thermoplastic material TOPAS COC 8007 is used herein because of its high mechanical rigidity, low water absorption (0.01%), and good optical transmissivity (91%) for optical observation [35]. The COC-based micromodel fabrication includes three major processes: micromold fabrication, hot embossing, and bonding. As shown in Figure 2a, the micromold is first fabricated using the standard SU-8 photolithography process. Figure 2b shows the SEM image of the microcavity arrays on the SU-8 micromold. 

The SU-8 micromold is assembled to a custom-machined micromold holder and fixed to a hot embosser (Ray Cheng Enterprise Co. Ltd., Taoyuan, Taiwan). Because the glass-transition temperature (*T*_g_) of COC is 78 °C, the COC substrate is placed above the SU-8 micromold and close to the hot embosser to preheat the COC substrate at 77 °C for 5 min. Then, a hot-embossing temperature of 83 °C (5 °C above *T*_g_) and an embossing pressure of 1.45 MPa are applied for 7 min. After cooling down to 70 °C for 15 min, the hot embosser is opened and the inversely embossed COC replicas are removed when one hot-embossing process has been completed. The embossed COC microchannel layer is bonded to another COC cover layer to enclose the microchannel. Because of a high-porosity micromodel layout, the microchannel hydraulic resistance is high; therefore, a high bonding-strength polymer-bonding method is required. In this paper, we select the high bonding-strength UV/Ozone method to seal the COC substrate [29]. The bonding procedure starts with predrilling another COC cover layer with 1.5-mm-diameter reservoir holes prior to bonding. The embossed COC microchannel is placed and the cover layers are treated in the UV/Ozone cleaner (PSD-UV, Novascan Technologies, Ames, IA, USA) for 300 s. The top surface of the COC layers is activated by UV/Ozone treatment for consequent low-temperature UV/Ozone bonding. The COC pair is aligned and put in the hot embosser press-bond at 3.7 MPa and 70 °C for 110 min. After UV/Ozone bonding, surgical needles are inserted into the cover plate reservoirs as the inlet and outlet connections. Figure 2c shows the porous micromodel after bonding and the surgical-needle insertion; all microchannels and microcavity arrays are well-bonded without de-bonding or fluid leakage. 

### 3.2. Microchannel Surface Wettability Characterization

**Figure 3:**
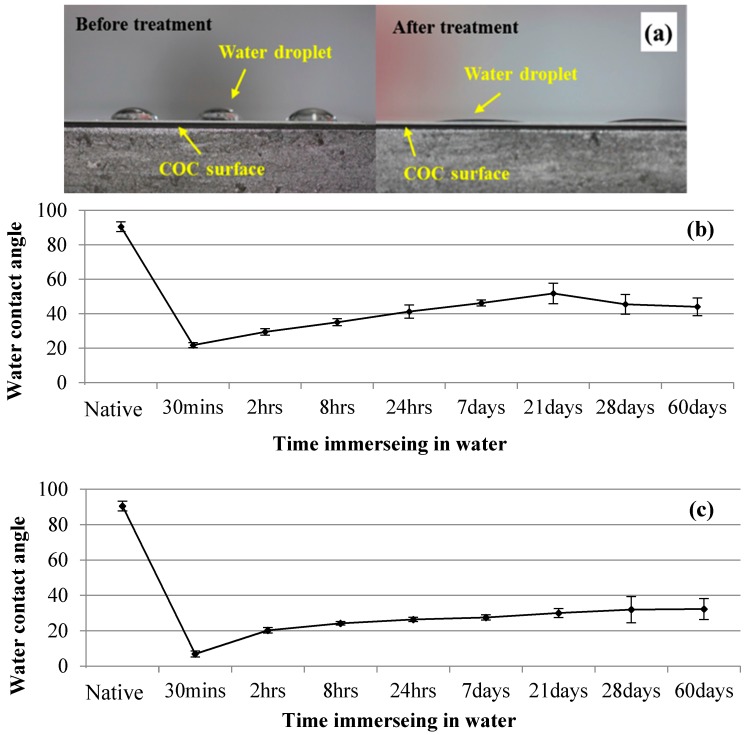
(**a**) Change in the COC-surface wettability after UV/Ozone treatment. The COC-surface water contact angle at different water-immersion times after (**b**) 100 s and (**c**) 300 s of UV/Ozone surface treatments.

One of the major challenges in using a polymer micromodel for two-phase-flow studies on natural porous materials is the wettability of the polymer. It is important to have a uniform and stable wettability throughout the pore space. In this study, we use UV/Ozone to physically treat the COC surface in order to enhance the bonding as well as to render the COC surface hydrophilic. UV/Ozone is widely used in dry cleaning aimed at removing organic contaminants from silicon surface. The mercury lamp outputs a 184.9 nm wavelength generating ozone in the chamber and a 253.7 nm wavelength that decomposes the ozone molecules. The continuous process of formation and decomposition of ozone molecules leads to surface oxidation and changes the COC-surface wettability. After UV/Ozone treatment, the COC surface becomes super hydrophilic. As shown in Figure 3a, the native COC surfaces exhibit weak hydrophobicity. After UV/Ozone surface treatment, the COC surface becomes hydrophilic. In the UV/Ozone bonding process, as shown in Figure 2a, the top, bottom, and side wall surfaces of the unbonded microchannel remain hydrophilic after UV/Ozone binding. Therefore, we used UV/Ozone treatment to make the micromodel's surface wettability similar to that of natural porous materials. 

The wettability of a UV/Ozone-treated surface is a dynamic process that may vary with time when subjected to an aqueous environment. Figure 3b,c show the contact angle of the UV/Ozone-treated COC surface after immersion in water from 30 min to 60 days for 100 s and 300 s UV/Ozone treatment, respectively. The native COC-surface water contact angle is approximately 90.5° ± 2.8°. After 100 and 300 s of UV/Ozone treatment, the water contact angle on the COC surface changes drastically, thereby making the COC surface superhydrophilic. After UV/Ozone-treatment of the COC surface in water for 30 min, it still shows high hydrophilicity in both the 100-s (21.7° ± 1.4°) and 300-s (7.8° ± 1.6°) UV/Ozone-treatment cases. The water contact angle increases with the water-immersion time until it reaches a plateau. For the 100-s UV/Ozone treatment (Figure 3b), the water contact angle increases from 21.7° to 48.2° within the first week (seven days) and then remains constant at 45°–50° between Day 7 and 60. For the 300-s UV/Ozone treatment (Figure 3c), the water contact angle increases to 20.2° and 24.2° after 2 and 8 h of water immersion, respectively; furthermore, the COC-surface water contact angle reaches a plateau (27°–31°) after 8 h. Therefore, in the following porous micromodel experiments, the COC surfaces were treated for 300 s with UV/Ozone to bond the COC chip and enhance the bonding strength. After bonding, the microfluidic channels were rinsed with water for more than 8 h in order to ensure good contact-angle stability—i.e., a water contact angle of approximately 24°—which is analogous to those of natural porous materials. In our experiments, we made the micromodel hydrophilic and uniformly maintained it in the same state for a long period.

### 3.3. Results of the Two-Phase-Flow Micromodel Experiments

**Figure 4:**
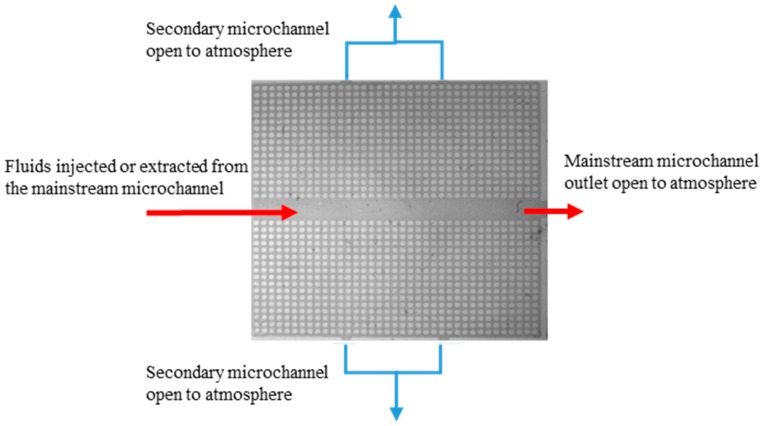
The inlet and outlets of the micromodel. The fluids are injected or extracted from the left inlet of the mainstream microchannel, which provides a constant-flow-rate boundary condition. The five outlets are connected to atmospheric air, thereby providing constant-pressure boundary conditions. The outlet on the right end of the micromodel is directly connected to the mainstream microchannel, and the other four outlets are connected to the top and bottom of the secondary microchannels (microarrays). The direction shown in the figure is for the injection experiments, and its opposite direction is for the extraction experiments.

We performed the experiments in micromodels comprising mainstream channels with different widths and different injection and extraction rates. The two-phase-flow experimental setup shown in Figure 4 involves imbibition and drainage processes. The experimental fluids are water and oil (gasoline). DI water is dyed blue to enhance visual observation and facilitate image-analysis of saturation. The dynamic viscosities of water and oil are 0.089 and 0.06 Pa·s, respectively. The surface tensions of the water and oil are 71.5 and 22.4 dyne/cm, respectively. Although the surface tension between oil and water is not measured directly, it is around 30–40 dyne/cm based on previous studies [36,37]. In the drainage process, the micromodel with a hydrophilic surface is first filled with dyed water. We drain the water out of the micromodel using two different methods: (1) injecting air into the model and (2) extracting water from the model. Since our syringe pump can provide a constant extraction rate, the extraction rates of both water and air are constant. In the imbibition process, the micromodel is first filled with oil. Then, the oil is extracted by injecting dyed water into the model. The constant injection and extraction rates of oil and water for both drainage and imbibition are 50, 100, 200, and 400 μL/min. During the drainage and imbibition processes, the high-speed camera records the dynamics of the fluid distributions. Injection or extraction is terminated when the fluid distribution reaches equilibrium.

#### 3.3.1. Water–Air Displacement: Air Injection and Water Extraction 

**Figure 5:**
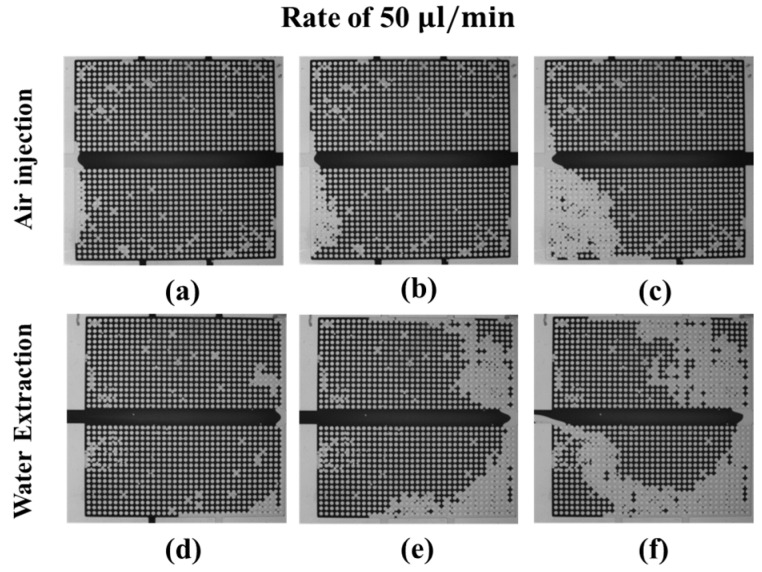
Dynamics of the water (dark)–air (transparent) distributions during the drainage process at an air-injection (**a**–**c**) and a water-extraction (**d**–**f**) rate of 50 μL/min; (**a**,**d**) correspond to the beginning, (**b**,**e**) to the middle, and (**c**,**f**) to the end of the drainage process.

**Figure 6:**
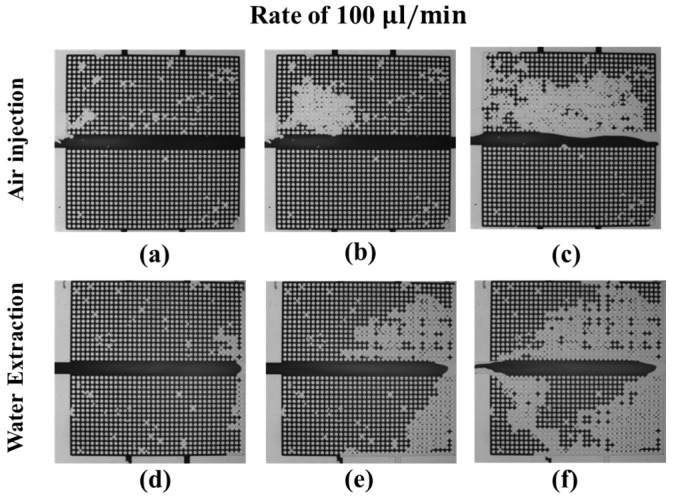
Dynamics of the water (dark)–air (transparent) distributions during the drainage process with an air-injection (**a**–**c**) and a water-extraction (**d**–**f**) rate of 100 μL/min; (**a**,**d**) correspond to the beginning, (**b**,**e**) to the middle, and (**c**,**f**) to the end of the drainage process.

**Figure 7:**
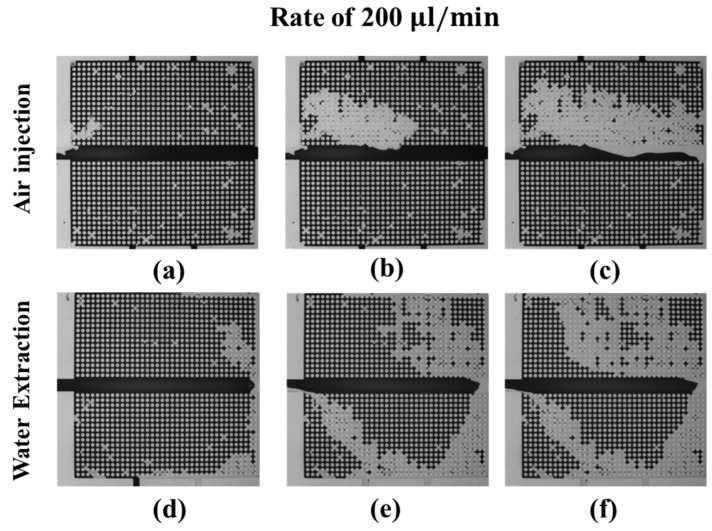
The dynamics of the water (dark)-air (transparent) distributions during the drainage process at an air injection (**a**–**c**) and water extraction (**d**–**f**) rate of 200 μL/min. (**a**,**d**) correspond to the beginning of the drainage process, (**b**,**e**) the middle of the drainage process, and (**c**,**f**) the end of the drainage process.

**Figure 8:**
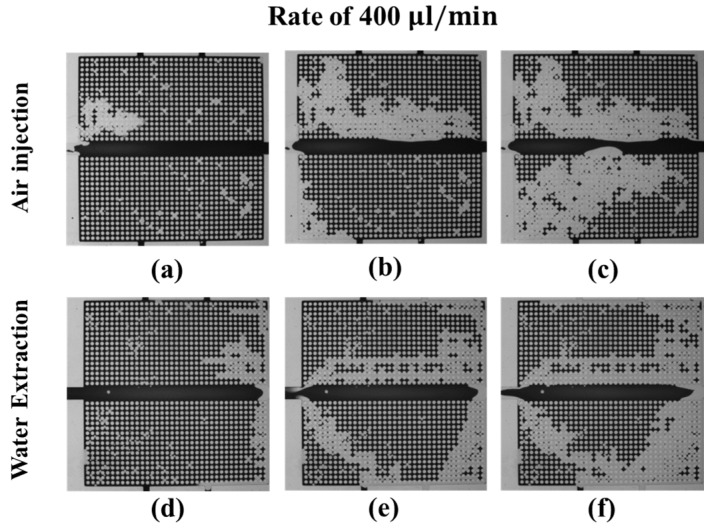
Dynamics of the water (dark)–air (transparent) distributions during the drainage process at an air-injection (**a**–**c**) and water-extraction (**d**–**f**) rate of 400 μL/min; (**a**,**d**) correspond to the beginning, (**b**,**e**) to the middle, and (**c**,**f**) to the end of the drainage process.

The water-drainage experiments are performed in two different processes: air injection and water extraction. Figure 5, Figure 6, Figure 7 and Figure 8 show the changes in the water–air distributions during the drainage process at air-injection and water-extraction rates of 50, 100, 200, and 400 μL/min. As shown in Figure 5a–c, the air gradually pushes out the dyed water in the bottom microarray. At the end of the experiment, the injected air bypasses most of the water and directly flows out of the model via the bottom outlet close to the left inlet, as shown in Figure 5c. For water extraction from the left inlet, the air first moves into the micromodel via the right outlet and quickly forms an air path connecting the right and bottom outlets, as shown in Figure 5d. In the middle of water extraction, air flows into the micromodel from the right, top, and bottom outlets. At the end of the experiment, all the air from the outlets flows out via the water extraction inlet, and the water distribution in the porous micromodel becomes steady, as shown in Figure 5f.

In Figure 6a–c, air pushes out the dyed water in the upper microarray. When the injected air flows out via the right outlet, the water distribution in the upper microarray quickly reaches a steady state. At the end of the experiment, the injected air pushes out most of the water in the upper microarray, as shown in Figure 6c. For water extraction from the left inlet, the air first moves into the micromodel via the right outlet and drains the upper microarray and the lower microarray, as shown in Figure 6d. In the middle of water extraction, the air uniformly drains the water in the upper microarray and forms a preferential pathway connecting the right and bottom outlets in the lower microarray. At the end of the experiment, all the air flows out of the micromodel via the water extraction inlet, and the water distribution in the micromodel becomes steady, as shown in Figure 6f.

In Figure 7a–c, the air pushes out the dyed water in the upper microarray. When the injected air flows out via the right outlet, the water distribution in the upper microarray quickly reaches a steady state. At the end of the experiment, the injected air pushes out most of the water in the upper microarray, as shown in Figure 7c. For water extraction from the left inlet, the air first moves into the micromodel via the right outlet and drains the upper microarray and the lower microarray, as shown in Figure 7d. In the middle of the water extraction, the air uniformly drains the water in the upper microarray, and the air forms a preferential pathway connecting the right and bottom outlets in the lower microarray. At the end of the experiment, all the air from the outlets flows out via the water extraction inlet, and the water distribution in the micromodel becomes steady, as shown in Figure 7f.

In Figure 8a–c, the air pushes out the dyed water in the upper microarray and then the lower microarray. When the injected air flows out via the right outlet, the water distribution in the upper microarray quickly reaches a steady state; then, the air pushes water in the bottom microarray. At the end of the experiment, the injected air pushes out more than 50% of the water in both the upper and lower microarrays, as shown in Figure 8c. For water extraction from the left inlet, the air first moves into the micromodel via the right outlet and drains the upper microarray and the lower microarray, as shown in Figure 8d. In the middle of the water extraction, the air uniformly drains the water in the upper microarray and forms a preferential pathway connecting the right and bottom outlets in the lower microarray. At the end of the experiment, all the air from the outlets flows out via the water extraction inlet, and the water distribution in the micromodel becomes steady, as shown in Figure 8f.

**Figure 9:**
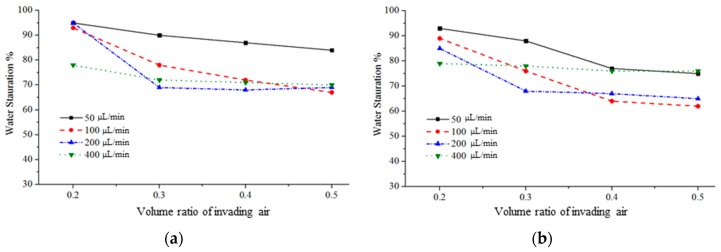
Water saturation versus the volume ratio of invading air in the models with (**a**) different air injection rates and (**b**) different water extraction rates.

Figure 9 shows the evolution of water saturation versus the volume ratio of the injected air, which is the ratio of the total injected air volume to the total pore volume of the micromodel. Because during the water extraction, air also moves out of the micromodel via the inlet, the amount of water extracted cannot be estimated only from the extraction rate of the syringe pump. Nevertheless, we can estimate the injected-air volume during the water-extraction experiments. Therefore, we use the volume ratio of the injected air for all experiments, as shown in Figure 9. For air injection, the water-saturation-decreasing rate increases with the air-injection rate. At injection rates higher than 100 μL/min, the residual-water saturations all converge to approximately 70%. For water extraction, the water-saturation-decreasing rate increases with the air-injection rate. Nevertheless, at the extraction rate of 400 μL/min, the water distribution reaches an equilibrium quickly, with water saturation of approximately 80%. This is because the preferential air path is readily formed at the beginning of the experiments. 

Both the air-injection and water-extraction experiments indicate that at injection/extraction rates of less than 200 μL/min, the minimum water-saturation rate decreases with increasing injection or extraction rate, as shown in Figure 9. However, at injection/extraction rates of 400 μL/min, our experimental results show that an increase in these flow rates does not significantly reduce the minimum water-saturation rate. Conversely, an increase in the water-extraction rate increases the minimum water-saturation rate. Therefore, we conclude that an increase in the air-injection rate can lead to a decrease in the minimum water-saturation rate; however, this is not a monotonic trend in water-extraction experiments because of the preferential path. 

**Figure 10:**
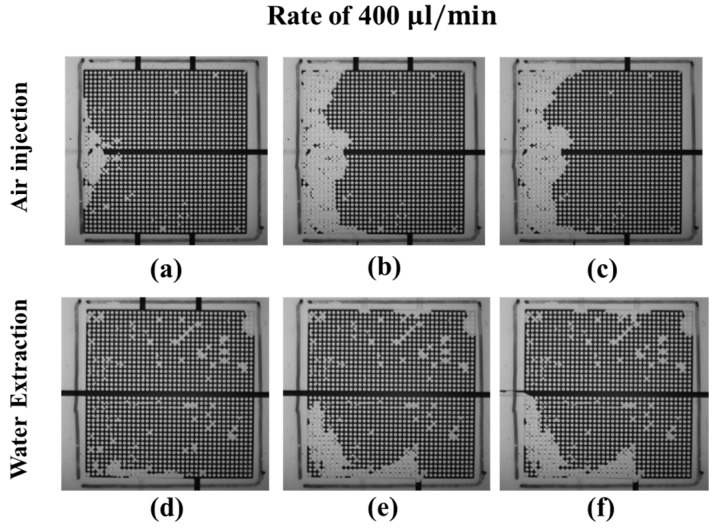
Dynamics of the water (dark)–air (transparent) distributions during the drainage process at an air-injection (**a**–**c**) and water-extraction (**d**–**f**) rate of 400 μL/min in a micromodel comprising a narrow mainstream channel with a width of 1 mm; (**a**,**d**) correspond to the, (**b**,**e**) to the middle, and (**c**,**f**) to the end of the drainage process.

Figure 10 shows the changes in the water–air distributions during the drainage process at an air-injection rate of 400 μL/min in the micromodels comprising narrow mainstream channels. In Figure 10a–c, the air uniformly pushes out the dyed water in the upper and the lower microarrays. When the injected air flows out via the left upper and bottom outlets, the water distribution in the microarray gradually reaches a steady state. At the end of the experiment, the injected air pushes out one-third of the water in both the upper and lower microarrays, as shown in Figure 10c. 

The effect of the width of the mainstream channel on the water drainage process is significant, as indicated by a comparison of Figure 8 and Figure 10. In the model comprising a narrow mainstream channel (Figure 10), the water is pushed out from the mainstream channel and the upper and lower microarrays at the same time because of the small difference between the widths of the mainstream and the microchannel. Since the water is displaced first in the narrow mainstream, the injected air can be connected in the upper and lower microarrays that leads to a uniform sweep. Therefore, the injected air is connected in the upper and lower microarrays. On the other hand, the air pushes water from the upper microarray first and then from the bottom microarray in the model comprising a wide mainstream channel (Figure 8). Nevertheless, the water in the wide mainstream channel is not displaced by air. The air in the upper and lower microarrays is disconnected; therefore, the air–water distribution patterns are totally different in the upper and lower microarrays. For water extraction at a rate of 400 μL/min, the air is pulled via all outlets into the micromodel comprising the wide mainstream channel. Once the air from the bottom is connected to the left inlet, the extraction process reaches equilibrium. In the narrow mainstream channel, the air does not flow through the main channel and less oil is extracted. 

#### 3.3.2. Oil-Water Displacement: Water Injection

**Figure 11:**
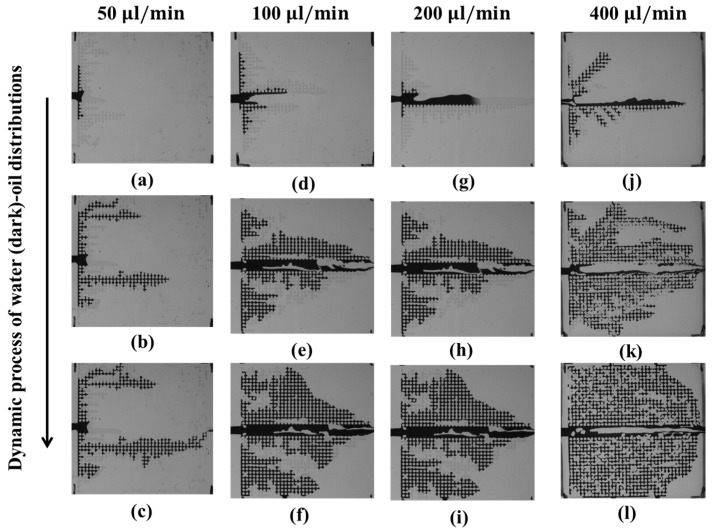
Dynamics of the water (dark)–oil (transparent) distributions during the imbibition process at water-injection rates of 50 μL/min (**a**–**c**), 100 μL/min (**d**–**f**), 200 μL/min (**g**–**i**), and 400 μL/min (**j**–**l**) in a micromodel. (**a**,**d**,**g**,**j**) correspond to the beginning; (**b**,**e**,**h**,**k**) to the middle; and (**c**,**f**,**i**,**l**) to the end of the imbibition process.

In the water-imbibition experiments, water is injected into the oil-filled micromodel. Figure 11 shows the oil–water distribution dynamics at different injection rates. The amount of oil extracted increases with the water-injection. Figure 11a–c shows that at an injection rate of 50 μL/min, a clear fingering water flow is formed in the lower microarray close to the mainstream channel. Water only flows in the newly formed preferential channel toward the upper and right outlets, and no more oil is extracted. When the injection rate increases to 100 μL/min, as shown in Figure 11d–f, the oil in the upper and lower microarrays is partially extracted but that in the mainstream channel mostly remains. As the water-injection rate increases to 200 μL/min, the injected water first pushes the oil in the mainstream channel and then pushes it to the upper and lower microarrays, as shown in Figure 11g–i. At a water-injection rate of 400 μL/min, the injected water pushes out most of the oil in the mainstream channel and the upper and lower microarrays, as shown in Figure 11j–l. However, the amount of residual oil in the mainstream channel is greater than that obtained at a water-injection rate of 200 μL/min. The oil-extraction efficiency in the mainstream channel (fracture) non-monotonically increases with the water-injection rate. 

From the air–water and oil–water experiments, we found that the injected air and water bypass the fracture, especially at the beginning of the experiments. The major reason for this bypass flow is the additional secondary outlets. In the classical pattern of the pore structure with fracture, the only outlet exists at the end of the fracture. In our new model, we added four secondary outlets to create an additional flow direction. Since the distance between the mainstream inlet and the secondary outlet is closer to the mainstream outlet, the pressure gradient is larger between the mainstream inlet and the secondary outlet than that between the mainstream inlet and the mainstream outlet. Therefore, even though the flow-resistance is larger in the secondary microchannel, the injected air tends to flow toward the secondary microchannel (top or bottom) first. At low-flow rates in the air-injection experiments, air flows out via the secondary outlet. However, when the air-injection rate increases, all the air flows toward the secondary microchannel first and then toward the mainstream outlet instead of flowing out via the secondary outlet, which becomes a flow pathway with lower resistance. In the water extraction experiments, because of the high pressure gradient between the mainstream inlet and the secondary outlet, the air flows into the secondary microchannel via the secondary outlet first and then through the mainstream outlet. A similar phenomenon occurs in the oil–water experiments.

## 4. Conclusions

The development of porous micromodels is important for visualizing two-phase-flow phenomena in natural porous materials. Compared with glass-based or other silicon-based micromodels, the thermoplastic micromodel demonstrated in this study provides the advantages of design flexibility, precision scales, low cost, and low fabrication complexity. The COC porous micromodels were herein fabricated via hot embossing followed by bonding. When using thermoplastics in a porous micromodel, the issues of high hydraulic resistivity and surface hydrophobicity need to be considered. In this research, we used the UV/Ozone bonding method to increase the bonding strength and to render the surface hydrophilic (analogous to the surface wettability of natural geomaterials) for successful porous micromodel preparation. 

The porous micromodel two-phase-flow experiments conducted herein show that, during the drainage experiment, the extraction efficiency upon direct water-extraction is higher than that obtained via air-injection. At low air-injection rates, the drainage process quickly reaches equilibrium (no more water is extracted) once the preferential channel connects the inlet and outlet. When the air-injection rate increases, the water-extraction rate increases and the saturation rate of the residual water decreases. The same situation occurs when we inject water to extract oil. Our experimental results show that the water- and oil-extraction efficiency in a fractured porous medium depends on the boundary conditions, the injection and extraction rates, and the width of the mainstream channel. In addition, the dynamics of the displaced water and oil in the mainstream channel are more complicated than those in the microarrays. Because of the complex two-phase-flow dynamics in the mainstream channel, the extraction efficiency in the mainstream channel does not monotonically increase with the injection rate. The common assumption that water or oil is extracted first in the fracture during the drainage and imbibition processes is not supported by our micromodel experiments.
